# Epidemiology, Diagnosis and Management of Extra-Pulmonary Tuberculosis in a Low-Prevalence Country: A Four Year Retrospective Study in an Australian Tertiary Infectious Diseases Unit

**DOI:** 10.1371/journal.pone.0149372

**Published:** 2016-03-10

**Authors:** Simon Pollett, Pamela Banner, Matthew V. N. O’Sullivan, Anna P. Ralph

**Affiliations:** 1 Marie Bashir Institute for Infectious Diseases and Biosecurity, University of Sydney, Sydney, NSW, Australia; 2 NSW Tuberculosis Program, Sydney, NSW, Australia; 3 Centre for Infectious Diseases and Microbiology, Westmead, Sydney, NSW, Australia; 4 Global and Tropical Health, Menzies School of Health Research, Darwin, Northern Territory, Australia; 5 Department of Medicine, Royal Darwin Hospital, Darwin, Northern Territory, Australia; Chinese Academy of Medical Sciences and Peking Union Medical College, CHINA

## Abstract

**Objectives:**

Extra-pulmonary tuberculosis (EPTB) is relatively neglected and increasing in incidence, in comparison to pulmonary tuberculosis (PTB) in low-burden settings. It poses particular diagnostic and management challenges. We aimed to determine the characteristics of EPTB in Western Sydney, Australia, and to conduct a quality assurance investigation of adherence to guidelines among Infectious Diseases (ID) practitioners managing EPTB cases.

**Methods:**

All adult EPTB cases managed by a large ID service during 01/01/2008–31/12/2011 were eligible for inclusion in the retrospective review. Data were extracted from patient medical records on demographic, diagnostic, clinical and management details, and on clinician adherence to local and international TB guidelines.

**Results:**

129 cases managed by the ID service were identified, with files available for 117. 98 cases were managed by the Respiratory service and were excluded. 98.2%(112/114) had been born in a country other than Australia. HIV status was tested or previously known in 97 people, and positive in 4 (4%). Microbiological confirmation was obtained in 68/117 (58.1%), an additional 24 had histopathological findings considered confirmatory (92/117, 78.6%), with the remainder diagnosed on clinical and/or radiological grounds. Median time to diagnosis post-migration from a high TB-burden country was 5 years (range 0–41). 95 cases were successfully treated, 11 cases defaulted, refused therapy or transferred, 2 cases relapsed and outcomes unknown or pending in 9 cases. No deaths occurred in the sample analysed. Clinician adherence to guidelines was high, but with scope for improvement in offering testing for co-infections, performing eye checks, monitoring blood glucose in patients receiving adjunctive corticosteroids, and considering drug interactions.

**Conclusions:**

Despite excellent TB outcomes in this setting, the low proportion of cases with susceptibility data is worrying in this era of increasing drug resistance, and illustrates the diagnostic difficulties faced even in a well-resourced setting. Vigilance for EPTB needs to remain high in those moving from high prevalence countries to Australia, even decades after immigration.

## Introduction

Australia, like many other high-resource settings, achieved a low incidence of tuberculosis (TB) in the latter 20^th^ century [1]. However, TB still poses important diagnostic and management challenges in Australia. Rates are approximately stable at 6 incident cases per 100,000 in 2009, the majority of which occurred in people born in TB-prevalent countries [2,3].

The conventional focus of TB programs has been on pulmonary TB (PTB) due to its transmissibility. Yet extra-pulmonary TB (EPTB) makes up over 40% cases in Australia and more than half when EPTB definitions include concurrent PTB co-infection [2]. This proportion has been reportedly increasing in Australia and many Western regions [2,4,5]. Furthermore EPTB has diverse manifestations which may mimic other pathologies [6] making it more diagnostically challenging, more frequently associated with diagnostic delay [7], and giving it greater potential for morbidity and mortality, particularly tuberculous meningitis (TBM) [8] when compared to PTB. Thus EPTB deserves an increasing focus.

A detailed characterisation incorporating socio-demographic, clinical, laboratory and radiological features of Australian adult EPTB cases is lacking. In particular, it remains unclear what level of evidence (including molecular and histopathological evidence) supports EPTB diagnoses in Australia. Such an assessment is required, particularly as the most recent National Strategic Plan for TB Control identified as a core priority the provision of a high standard of diagnosis [1].

Maintaining quality treatment practices for TB in Australia is another key goal [1]. TB Control Programs in Australia generally report highly successful outcomes, with death being uncommon (recent death rates due to TB are reported as 1.1 to 1.5%), local transmission generally limited and relapse after treatment being very rare [2,9]. However, a clear understanding of the EPTB management practices of Infectious Diseases clinicians in this country is lacking. Low disease prevalence might, in well-resourced settings, be paradoxically associated with suboptimal detection and management. Such an assessment would allow appraisal and potentially improvement of Australian standards of EPTB care, and provide the basis for development of tailored EPTB management guidelines for this and other low-prevalence regions.

Thus, the first objective of this study is to describe the socio-demographic, clinical and laboratory characteristics of EPTB cases presenting to a large tertiary Australian Infectious Diseases (ID) unit for care, particularly the level of supporting evidence behind EPTB diagnoses. The second objective is to describe EPTB management practices, treatment outcomes and adverse treatment effects of EPTB cases managed in this ID unit; and in particular to compare management practices against those recommended in local and international guidelines.

## Materials and Methods

### Setting and study population

The Centre for Infectious Diseases and Microbiology (CIDM) provides tertiary-level inpatient and ambulatory care for the Western Sydney Local Health District (WSLHD), New South Wales (NSW). WSLHD services an urban population of 830,000 persons, 40% of whom were born overseas and fewer than 2% of who are Aboriginal Australian [10]. The estimated TB incidence rate is higher in Western Sydney than the national average at 11/100,000 [11].

In Australia TB cases are generally managed by public inpatient or ambulatory tertiary ID or Respiratory Medicine units. Directly observed therapy (DOT) is mandated by the Australian Government in certain states, including NSW [12], where the majority of annual Australian TB cases are reported [2]. Throughout Australia, assessment and treatment of TB is provided free of charge, irrespective of residency status [13]. NSW clinicians have free access to national peer-reviewed antimicrobial treatment guidelines [14], State Government policies on EPTB management principles [15] and international TB treatment guidelines provided by the Centre for Disease Control and Prevention (CDC) [16] and the World Health Organisation (WHO) [17].

### Inclusion criteria

Adults with EPTB, with or without PTB, diagnosed and managed at CIDM as ambulatory or admitted patients between 1^st^ January 2008 and 31^st^ December 2011 were included. EPTB was defined as a diagnosis based on “one culture-positive specimen, histological or strong clinical evidence consistent with active extra-pulmonary disease, followed by a decision by a clinician to treat with a full course of antituberculous chemotherapy”, in accordance with WHO definitions[17] We also included diagnoses made on molecular positive results alone, in appropriate clinical contexts. Cases were identified by screening hospital separation data and WSLHD TB clinic databases. Due to finite study resources, adult EPTB cases managed by WSLHD Respiratory Medicine units were excluded. In order to minimize selection bias, mortality data from these Respiratory Medicine managed cases, and cases which died before referral to either the Respiratory Medicine or ID Unit, were collected from aggregate mortality metadata maintained by the WSLHD TB service.

### Study procedures, including definitions

Data on socio-demographic, clinical, radiological, histopathological, microbiological and other laboratory variables were collected from patients’ medical files. Data on the management practices by treating clinicians, treatment outcomes and adverse events were also collected from the medical file at 6–12 months after the study period concluded. Data were collected on key management practice quality indicators generated from local, national and international guidelines (S1 Table).

‘Years in Australia’ was derived from self-reported year of immigration to Australia. Anatomical site of disease was determined by radiographic, microbiological, histopathological and/or clinical evidence. ‘Miliary TB’ referred to cases with characteristic chest imaging findings. Chest x-ray or computed tomographic (CT) findings ‘suggestive of active PTB’ were defined as consolidation, infiltrates, miliary pattern, or cavitation consistent with PTB, but excluded findings which might indicate past infection only (fibrosis, calcification). ‘Confirmed pulmonary co-infection’ was defined as microbiological positivity (microscopy or culture or molecular result consistent with *Myocbacterium tuberculosis* complex) on sputum, broncho-alveolar lavage or lung biopsy specimen.

‘High risk patients’ for Hepatitis B virus infection (defined as Hepatitis B surface antigen positivity) or Hepatitis C virus seropositivity was defined as those born in Africa, Asia, the Pacific Islands or those with a recorded history of injectable drug use. ‘Iatrogenic immunosuppression’ was defined as the use of systemic steroids, calcinuerin inhibitors, mycophenylate, alkylating agents, biological agents known to be a risk factor for tuberculosis reactivation, or chemotherapy. ‘Immunocompetent’ was defined as the absence of HIV, diabetes, malignancy or iatrogenic immunosuppression. A ‘potentially interacting medication combination’ was defined as those medications interacting with rifampicin via the CYP450 pathway, including anticonvulsants, cyclosporine, dapsone, implantable/oral contraception, methadone, non-nucleoside reverse transcriptase inhibitors, protease inhibitors, sulfonylureas and warfarin.

‘Any positive microbiology’ included a positive microscopy, culture or molecular result consistent with *M*. *tuberculosis* or *M*. *bovis*. Direct acid-fast bacilli (AFB) detection was performed using auramine staining with confirmation of positives using Kinyoun staining. Mycobacterial cultures were performed both by a mycobacterial-growth indicator tube liquid culture system [BACTEC MGIT 960, Becton Dickinson] and culture on Lowenstein-Jensen solid media. Samples from superficial sites (skin, joint fluid, lymph nodes) were additionally cultured on blood agar for detection of *M*. *haemophilum*. Confirmation of cultured species was by HPLC of extracted mycolic acids, and antigen detection using the SD Bioline TB Ag MPT64 Rapid Assay (Standard Diagnostics, Gyeonggi-do, Republic of Korea) in addition to an in-house probe specific real time PCR targeting IS6110 with a sequence specific probe [18]. Drug susceptibility testing was performed phenotypically and/or by *rpoB* gene sequencing for rifampicin resistance mutations and/or line probe assay for rifampicin and isoniazid resistance determinants (GenoType® MTBDRplus, Hain Lifescience, Nehren, Germany). Histopathological examination included eosin & haematoxylin stains and Ziehl-Neelsen stains for acid-fast bacilli. ‘Positive histopathology’ was defined as presence of any granulomata, acid-fast bacilli, or giant cells. The interferon gamma release assay (IGRA) used at CIDM over the study period was the Quantiferon Gold In-Tube test [Cellestis Limited, Carnegie, Victoria, Australia].

A ‘standard first line regimen’ was defined as four-drug therapy containing isoniazid, rifampicin, ethambutol and pyrazinamide, generally four drugs for two months followed by isoniazid and rifampicin for a continuation phase. ‘Directly observed therapy’ (DOT) was defined as regularly supervised antituberculous therapy administration by WSLHD TB clinic staff, including watching the patient swallow their medications, monitoring the patient for side-effects and general health or other concerns, liaising with a physician regarding any problems and documenting all this, every day except weekends and public holidays. A ‘treatment adverse event’ was defined as the new occurrence of an abnormality known to be associated with anti-TB treatment which developed after commencing treatment. Outcomes were categorised as successfully treated, relapsed, defaulted, transferred or died according to WHO definitions [19].

An ‘absolute indication for steroid therapy’ was defined as TB meningitis or pericardial disease. ‘Steroid-induced hyperglycaemia’ was defined as random blood or office/bedside glucometer values of glucose > 7.7 mmol/L in a non-diabetic whilst on steroids. Upper limits of normal for alanine transaminase (ALT) and bilirubin were defined as 35 U/L and 22 μmol/L respectively [20]. ‘Treatment-related hepatotoxicity’ was defined as a new elevation of ALT or bilirubin above the upper limits of normal while on anti-tuberculous therapy. ‘Inflammatory response’ was defined as a documented clinical impression of any paradoxical inflammatory reaction to TB therapy.

### Data management and statistical analysis

Data were entered into an Access 2007 customised database. Continuous variables were summarised as mean and/or median and range or interquartile range, as appropriate. These statistics, with frequencies of categorical and binomial variables, and Chi or Fisher’s exact tests to compare independent proportions, were calculated using Stata version 13 (Stata Corp, College Station, TX, USA)

### Ethics

This study was approved by the Ethics Committee of the Western Sydney Local Health District (WSLHD) as a quality assurance activity. As such, written informed consent was not obtained by participants for their clinical records to be used in this study. Patient records/information was de-identified prior to analysis.

## Results

Between January 1^st^ 2008 and December 31^st^ 2011, 129 adults with EPTB were assessed and managed at CIDM. 117 were included in the study, with the files unavailable for the remaining 12. Ninety-eight EPTB cases were assessed and managed by WSLHD respiratory medicine during the same study period and were not included in the study. Two EPTB cases died before referral to either the Respiratory Medicine or ID service. Table 1 presents the socio-demographic characteristics of the cases included in the study. Almost all (98.2% [112/114]) had been born in a country other than Australia.

**Table 1:** Sociodemographic characteristics of EPTB cases.^a^

	*n/N*	%
Total	117/117	100
Country of birth		
South Asia^b^	67/114	58.8
East and South East Asia	27/114	23.7
Middle East	8/114	7.0
Africa	5/114	4.4
Europe	3/114	2.6
Australia	2/114	1.8
Pacific Islands	1/114	0.9
Latin America	0/114	0.0
Sex		
Male	62/115	53.9
Female	53/115	46.1
English as primary language	82/113	72.6
Permanent Australian resident status	82/110	74.5
	Median (range)	
Years in Australia	5 (0–41)	
Age in years	36 (20–80)	

^a^Numbers may not add up to total due to missing values

^b^India, Pakistan, Bangladesh, Nepal, Sri Lanka

### Clinical and laboratory characteristics

Table 2 presents the clinical characteristics of the EPTB cases studied. Co-existent probable or confirmed PTB was present in 17.1% (20/117), with no cavitating disease seen. In those who recalled a history of previous TB, 5/9 recalled a PTB history, 3/9 an EPTB history and one could not recall the primary site of disease. Country of prior treatment was Australia in 3/9, another country in 5/9 and unknown in one. Co-morbidities were relatively uncommon (Table 2). Of the four HIV co-infections, median CD4 count at the time of TB diagnosis was 116 cells /mL (range 19–134 cells /mL).

**Table 2:** Clinical characteristics and laboratory evidence of EPTB cases.^a^

Clinical characteristics	*n/N*	%
Total	117/117	100.0
Site of disease		
Lymph node	63/117	53.8
Gastrointestinal	18/117	15.4
Central Nervous System (excluding eye)	9/117	7.7
Bone	9/117	7.7
Genitourinary	9/117	7.7
Miliary	7/117	6.0
Skin	6/117	5.1
Pleura	4/117	3.4
Pericardial	4/117	3.4
Eye	3/117	2.6
Other^b^	7/117	6.0
Co-existing pulmonary disease (probable)	14/117	12.0
Co-existing pulmonary disease (confirmed)	6/117	5.1
Multi-site	28/117	23.9
History of previous tuberculosis disease	9/113	8.0
Co-morbidities and co-existing medical conditions		
Chronic kidney disease	5/113	4.4
Iatrogenic immunosuppression	4/113	3.5
HIV^c^	4/95	4.2
Diabetes	7/113	6.2
Alcohol abuse	0/113	0.0
Injectable drug use history	2/113	1.8
Malignancy	3/113	2.7
Hepatitis B virus infection^d^	6/86	7.0
Hepatitis C virus seropositive^e^	5/70	7.1
Potentially interacting medications	18/112	15.9
Laboratory evidence of diagnosis^f^	*n/N*	%
Smear positive	8/117	6.8
Culture positive	66/117	56.4
PCR positive	36/117	30.8
Any microbiological evidence	68/117	58.1
Histopathology positive	66/117	56.4
Any microbiological or histopathological evidence	92/117	78.6

^a^Numbers may not add up to total due to missing values

^b^Soft tissue abscess, nasopharynx, psoas, occult

^c^Includes one diagnosis made after TB diagnosis. Denominator = those with either previously documented HIV or who underwent HIV testing at time of TB diagnosis with test result available

^d^Denominator = those with either previously documented HBV or who underwent HBV testing at time of TB diagnosis with test result available

^e^Includes three diagnoses made after TB diagnosis. Denominator = those with either previously documented HCV or who underwent HCV testing at time of TB diagnosis with test result available

^f^Denominator is all EPTB cases (rather than those who had each respective test performed) to present what proportion of all cases had laboratory evidence of diagnosis and take into account physician diagnostic approaches and difficulty in obtaining adequate diagnostic specimens in some cases

Table 2 also presents the laboratory characteristics of the EPTB cases studied. *M*. *tuberculosis* disease was confirmed on culture in 56.4% (66/117) of cases. Of the 5.1% (6/117) cases with culture-positive respiratory samples, one case had normal chest imaging (HIV negative). Two EPTB cases (gastrointestinal, psoas) which were culture and smear negative had a positive molecular test. 24 additional people had histopathological findings consistent with TB; thus in total, 78.6% (92/117) were considered to show microbiological or histopathological evidence of EPTB. Of those patients with TB-consistent histopathology, 98% (65/66) showed granulomata, 62% (41/66) showed caeseating granulomata, and 5% (3/66) showed acid-fast bacilli. EPTB diagnoses in the remaining patients were based on radiological and/or clinical grounds.

When tested, IGRA was positive in 70% (21/30) of microbiologically or histopathologically confirmed ETB cases, and in 50% (5/10) of EPTB cases diagnosed on clinical/radiological grounds. However, there was no strong evidence that IGRA positivity was more common in those with laboratory evidence of TB than those without (p = 0.36). Of those with available drug-susceptibility testing results (by phenotypic or genotypic methods), 6% (4/66) had any drug resistance, 5% (3/66) had isoniazid resistance, 2% (1/66) had pyrazinamide resistance, 2% (1/66) had rifampicin resistance and none had multi-drug resistance.

### Management practices

The management practices of the treating clinicians are shown in Table 3. For baseline eye reviews, no alternative screen apart from referral to a formal ophthalmology clinic was described in the medical files. In those with a potential drug-drug interaction, there was documentation of acknowledgment +/- action taken in 58% (10/17) of cases. Of those cases which did not receive a standard first line regimen, 60% (6/10) had moxifloxacin substituted for ethambutol, 30% (3/10) had ethambutol omitted (with no substitute agent) and 10% (1/10) had rifampicin-pyrazinamide dual therapy prescribed. Regarding HIV-TB co-infection, 25% (1/4) of cases were on anti-retroviral therapy (ARV) at the time of TB diagnosis. In the remaining HIV positive patients, ARV was commenced at a median time of 5 days (range 3–11 days) after commencing TB therapy. In those cases with HIV co-infection 0% (0/4) were treated with rifabutin rather than rifampicin (with all 4 cases receiving efavirenz) and 100% (4/4) received daily TB therapy for at least the intensive phase of therapy. In those cases with an absolute indication for steroids, 100% (13/13) received it.

**Table 3:** Characterisation of clinician management practices.^a^

	*n/N*	%
Total	117/117	100
Baseline adjunctive investigations performed		
HIV serology performed^b^	94/112	83.9
HBV screening serology (in high risk patients)^c^	75/102	73.5
HCV screening serology (in high risk patients)^c^	63/104	60.6
Formal ophthalmology review (in cases on ethambutol)	29/96	30.2
Chest imaging performed (to consider pulmonary co-infection)	111/116	95.7
Baseline liver function indices, creatinine and FBC pre-treatment	110/112	98.2
Anti-tuberculous therapy		
Used a standard first line regimen	99/109	90.8
Referred for DOT	97/107	90.7
Daily dosing used for at least part of treatment	96/103	93.2
Daily dosing used for entire treatment	24/96	25.0
Median duration of therapy in months (range)^d^		
All cases	6.7 (5.5–16.2)	
Lymph node disease	6. 2 (5.6–12.3)	
Central nervous system disease	12.3 (8.0–16.2)	
Pleural disease	7.6 (5.8–11.4)	
Pericardial disease	7.2 (6.0–8.3)	
Gastrointestinal disease	7.1 (6.0–12.5)	
Genitourinary disease	8.9 (5.9–12.0)	
Bone disease	11.8 (6.0–13.1)	
Miliary disease	12.3 (9.0–16.2)	
HIV co-infection	11.3 (9.0–12.5)	
B6 supplementation (in cases receiving isoniazid)		
Any B6 supplementation used	102/103	99.0
25 mg dose of B6 commenced	97/102	95.1
Steroid administered^e^		
Any	24/111	21.6
Prednisone	19/111	17.1
Dexamethasone	6/111	5.4
Hydrocortisone	1/111	0.9
Indication for steroids		
CNS disease	9/24	37.5
Pericardial disease	4/24	16.7
Other^f^	11/24	45.8
BSL monitoring on steroids	9/18	50

^a^Numbers may not add up to total due to missing values

^b^Excluded those with a known history of HIV

^c^Excluded those with a known HBV or HCV infection respectively

^d^Excluded defaulters, those still on treatment and transfers. Estimates by disease site include multi-site disease

^e^Some cases received >1 type of steroid

^f^Gastrointestinal, miliary, genitourinary, node, paradoxical reaction, pleural, adrenal, eye and/or pulmonary TB

### Outcomes

Table 4 presents the outcomes and toxicities of the EPTB cases studied. The proportion of successfully treated cases (95/97, 97.9%) was derived using a denominator which excludes defaulters, those who transferred, those still on primary treatment and missing data. Including defaulters, missing data, those still on primary treatment and transfers in the denominator, the proportion of successful treated cases was 95/117 (81.2%). Of the two cases which later relapsed (one GI TB, the other nodal TB), both cases were microbiologically unconfirmed on initial diagnosis with relapse microbiological data unavailable. One of the cases was immunocompetent and otherwise well, and HIV co-morbidity data were not available in the other case. One of the two relapses occurred in a person who received DOT (DOT status was unknown in the other relapse). Relapse rates did not differ whether there was initial laboratory confirmation or not (p = 0.3), although given the low proportion of relapses overall this study would be likely underpowered to detect such a difference. Adverse events from anti-TB treatment were uncommon. Six patients defaulted from therapy, due to overseas travel in 2 (33%) and unknown reasons in the remainder. Of those who had a documented inflammatory response to TB treatment, 47% (9/19) were treated with steroids, and 11% (2/19) occurred in the setting of newly-commenced ARV. With respect to mortality, none of the 117 cases in the analytic sample died. From separate WLSHD aggregate mortality metadata collected for all TB cases in WLSHD during the study period, it was determined that 1 of the 98 EPTB cases managed by the Respiratory Unit died, as did two EPTB cases before referral to either the ID or Respiratory service, giving an estimated EPTB mortality rate of 3/229 (1.3%).

**Table 4:** Treatment outcomes and adverse reactions.

	*n/N*	%
Total	117/117	100
Treatment outcomes		
Still undergoing primary treatment	1/117	0.9
Successfully treated^a^	95/97	97.9
Relapsed^a^	2/97	2.0
Defaulted or refused therapy	6/117	5.1
Transferred out	5/117	4.3
Died	0/117	0.0
Outcome unknown (missing data)	8/117	6.8
Treatment toxicities^b^		
Hepatotoxicity		
ALT > ULN^c^	32/79	40.5
ALT > 3 x ULN^c^	10/79	12.7
ALT > 5 x ULN^c^	7/79	8.9
Bilirubin > ULN^c^^,^^d^	8/106	7.5
Retinal toxicity (possible)^e^	2/29	6.9
Inflammatory response syndrome	19/103	18.4
Steroid hyperglycaemia	3/9	33.3
Clinically evident neuropathy^f^	0/108	0.0

^a^Excluded defaulters, transfers and missing data

^b^Denominator size excludes missing values

^c^ULN = upper limit of normal, excludes those with elevated ALT pre-treatment

^d^Excludes those with elevated bilirubin pre-treatment

^e^In those receiving ethambutol and who underwent eye review

^f^Evolving in in those receiving isoniazid

## Discussion

We report for the first time a comprehensive characterisation of adult EPTB diagnoses and management by a large Infectious Diseases unit in Western Sydney, Australia. Importantly, we reveal the extent of diagnostic challenges even in a well-resourced setting, with only approximately half of cases being microbiologically confirmed and having susceptibility data available. This has important implications for selection of empirical treatment regimens in this era of increasing drug resistance. We show that vigilance for EPTB needs to remain high in immunocompetent people from high-burden settings. We also show that despite good overall adherence among practitioners to local and international TB management guidelines, certain areas of management, including testing for relevant co-infections, can be improved.

People with EPTB in this study were relatively young (median 36 years) and predominately overseas-born, particularly South Asia, followed by East and South East Asia (Table 1). While median time to EPTB presentation after immigration was 5 years, disease was seen up to four decades post-immigration. This highlights the need for considering EPTB as a differential diagnosis in overseas-born patients with compatible clinical features irrespective of their time living in a low-prevalence country. These sociodemographic characteristics (younger, overseas-born people and older local residents) are concordant with NSW and Australian TB surveillance data overall [2,3], with similar patterns in other low-prevalence countries [21].

The distribution of EPTB phenotypes was similar to that in local, state and national Australian TB surveillance data [2,3] and in studies from a range of high and low prevalence countries [5,21–24]. A lower than expected frequency of pleural disease was seen in this study, however, perhaps as pleural TB was more likely to be managed by respiratory physicians. Most patients had no detectable immunosuppression (85%, 96/113). The rate of HIV co-infection, a well-known risk factor for TB and particularly EPTB, was relatively low (4.2%), but higher than the national average of 1% of patients diagnosed with TB [2]. Diabetes was the single most common immunosuppressive illness (6.3%).

Over half (68/117, 58.1%) of all EPTB cases had supporting microbiological evidence by any method from any specimen, even with the use of liquid culture systems, molecular techniques and flurochrome staining. Molecular techniques provided an incremental yield over culture in only 2 cases. The high quality of culture in our setting may mean that the additional gain from molecular methods is less than has been reported from low-resource settings [25]. The proportion microbiologically confirmed is comparable with other studies of EPTB in developed regions such as France (58–61%), [26,27] and the US (67–76%) [5]. This likely reflects inherent difficulties in obtaining specimens from a non-pulmonary site and the relatively paucibacillary nature of EPTB [28].

Inclusion of cases positive by histopathology increased the overall proportion with laboratory evidence to over 75%. In this study, the pathognomonic finding of ‘caseating granuloma’ was absent in over a third of histopathology-positive cases. The non-specific granulomata seen in the remainder of histopathology-positive cases may also be seen in other infections (e.g. histoplasmosis) or non-infectious disorders (inflammatory bowel disease, sarcoidosis), and caution is needed in making a definitive EPTB diagnosis based on this, even with a high test pre-test probability of TB [29].

Around 2 in 10 EPTB cases were made on clinical and/or radiological grounds alone. That such a high proportion of cases are only presumptively diagnosed has implications for the accuracy of EPTB surveillance data in this region. To address this recognised problem, the National Australian TB strategy has recommended that future surveillance indicators include what proportion of new cases are culture-confirmed [1]. The development and uptake of standardized diagnostic scoring systems for various EPTB subtypes may be useful for the generalizability of EPTB surveillance and other EPTB studies in the future [30,31].

Only around half (56.4%, 66/117) of all EPTB cases had phenotypic or genotypic drug-susceptibility results available to guide therapy. Such a low proportion has implications for individual case management—and for control and surveillance of drug resistant TB in this region. Of those with susceptibility results, profiles were comparable to national surveillance data with a low overall prevalence of any drug resistance and isoniazid monoresistance as the leading pattern [32]. IGRA was negative in 30% of EPTB cases with microbiological or histopathological evidence, despite the relatively low rates of immunosuppression, highlighting that this is not a reliable diagnostic tool for active EPTB [33].

Chest imaging was performed in over 95% of EPTB cases, an important diagnostic and public health strategy given the relatively common frequency of coexistent pulmonary disease both in this study (microbiological proven PTB in 6%, 7/117) and in regional TB surveillance data [3]. Importantly, one of the microbiologically-proven PTB co-infections in this study had normal chest imaging and was culture negative on extra-pulmonary specimens. This has been described in other studies and highlights the need to consider obtaining induced sputa or other respiratory samples in all EPTB cases where possible, irrespective of chest imaging findings [34].

Over 15% of EPTB cases did not undergo HIV screening. Given the association between HIV and EPTB and that many TB cases are associated with migration from HIV high-prevalence countries, HIV screening should be performed in all EPTB cases. Increasingly-used default ‘opt-out’ systems of HIV screening may be useful for this purpose [35]. Screening for chronic HBV infection occurred in less than three-quarters of high-risk patients. A significant proportion of these EPTB cases originated from HBV-prevalent regions, and screening for HBV in such patients could facilitate a timely assessment of HBV before chronic sequelae occur. Moreover, a high HBV viral load independently predicts TB drug liver injury [36]. Knowledge of HBV serostatus could be used in the risk stratification for TB-therapy associated hepatotoxicity, and HBV serostatus should be known prior to the use of adjunctive corticosteroids. A similar argument can be made for improving the low HCV screening rates (60.6%) seen in this sample. IVDU is a risk factor for both HCV and active TB, and healthcare-associated HCV may occur in TB prevalent countries [37]. A notable number of cases (five) from this study were HCV co-infected. HCV infection may also predict development of hepatitis during TB treatment, even with normal baseline transaminases [38].

Of all adjunctive investigations recommended for TB care, eye reviews were the least implemented in this study. Less than a third of those on ethambutol underwent baseline eye review and this only occurred through a formal ophthalmological referral. In Australia, such referrals to formal ophthalmology services in the public system are often delayed due to very long waiting times [authors’ observation]. Implementing bedside screening practices using Ishihara and Snellen charts, as per international recommendations [16], could increase the rate of any eye review and ease burden on ophthalmological services.

Around 1 in 10 cases were treated without DOT, despite this being mandated in NSW [12]. This may reflect the increasing controversy as to the true effectiveness of DOT in low-prevalence settings [39]. While >90% of cases commenced therapy with daily dosing, one in five of these cases received daily therapy for the whole duration of therapy (Table 3).The efficacy of intermittent versus daily dosing regimens remains a controversial area in contemporary EPTB guidelines. While non-inferiority of intermittent dosing in most patient groups was demonstrated decades ago for pulmonary TB [40], it is still unclear if this can be generalised to EPTB. Although Australian TB guidelines recommend that HIV-negative PTB cases can receive three-times-weekly therapy after a minimum of 2 weeks daily therapy, these guidelines do not specify if such an approach can be generalised to EPTB disease [14]. CDC/ATS guidelines suggest that EPTB disease may be treated with intermittent dosing, although emphasise this is based on expert opinion[16]. All HIV co-infections received daily therapy for at least the intensive phase of TB therapy, and this approach is supported by a meta-analysis which demonstrates improved outcomes in HIV-TB co-infected patients receiving daily TB therapy [41]. No cases of HIV-TB co-infections received rifabutin to replace rifampicin, despite all co-infected cases receiving a rifampicin-interacting antiretroviral (efavirenz). Combination of these drugs is considered acceptable but where alternatives can be accessed, their use is encouraged.

All TB-HIV co-infected people not previously on ARV commenced ARV within 2 weeks of TB therapy, and two of these cases developed IRS which may have been related to such early ARV therapy. This is an evolving area in the literature with ongoing uncertainty as to the optimal time to commence ARV relative to TB therapy [42]. In general it has been recommended to commence between 2 to 8 weeks in those with CD4 counts under 200/mL [17]. Some evidence suggests a mortality benefit when treatment is commenced even earlier than 2 weeks in those with profound immunosuppression [43]. Importantly though, most of this literature is focused on PTB, and whether the findings can be generalised to EPTB requires further study [44].

There was considerable variation in durations of therapy, both between different sites of tuberculous disease and within sites of disease. Overall, treatment durations were in keeping with the CDC/ATS guideline for lymph node, gastrointestinal, pleural, pericardial and CNS disease, but longer than recommended for bony TB (in this study, median 11.8 month; recommended 9 months), genitourinary TB (median 8.9 months; recommended 6 months) and miliary TB (12.3 months; recommended 6 months)[16]. However, there is lack of consensus among guidelines on recommended treatment durations for different forms of EPTB, due to varying levels of evidence in this field [14,16,17] and the difficulties in defining cure. Also, many unmeasured factors may have contributed to clinical decisions about the final, individualised duration of therapy prescribed for these cases. Thus while there appears to be a tendency to over- rather than undertreat some forms of EPTB, it is not possible to comment on whether shorter treatment courses could have been successful.

Pyridoxine was used in nearly all people receiving isoniazid. Corticosteroid therapy was administered to all with TB meningitis and pericarditis, indications with moderate to high level evidence [45–47]. Testing for steroid-induced hyperglycaemia for those on steroids could be improved, however, particularly given the proportion found in this sample (3 of 9 tested). Moreover, a high prevalence of pre-diabetes has been observed in those presenting with TB to South Asian clinics in recent years [48] and any blood glucose measurement in such populations could be useful to detect undiagnosed insulin resistance.

Treatment outcomes (excluding defaults and transfers for which outcomes were unknown) were excellent overall. No mortality occurred in the analytic sample even though there was a significant proportion of CNS disease, a phenotype carrying a mortality of up to 25% in some studies from low-prevalence countries [22]. In order to minimize selection bias with respect to survival outcomes, we determined that one death occurred in cases excluded from analysis due to being managed by Respiratory Medicine, in addition to two cases which died before referral to either the ID or Respiratory Medicine service. Comparing cure rates to other low-prevalence countries is limited due to differences in comorbidities, EPTB phenotype distributions and denominator sizes.

Defaults and therapy refusal occurred in 6 individuals. Such defaults may increase the risk of drug resistance in Australia or other countries to which cases return. While this study was not designed or powered to determine risk factors for defaulters, travel to a country of home birth was a factor in 2 of 6 cases. Further studies over a longer period of time could clarify risk factors for default in our region, as part of a strategy to minimise defaults. Hepatitis was the leading adverse treatment event with over a third of patients having documented hepatitis on therapy. This frequency appears to be at the upper end of the range described in other TB studies, which ranges from 2.3% to 27.7% [24,49], although definitions of ‘hepatitis’ varied in some of this literature (we counted any elevation of ALT above normal as ‘hepatitis’), and detection depends on assiduousness of testing.

We excluded cases managed by Respiratory units which may limit the generalizability of our findings; however, the aim was to review practice among Infectious Diseases clinicians. The particular demography of WLSHD, including higher than average rates of being born overseas, and of TB-HIV co-infection, also requires consideration before generalising the study findings to a broader context. In particular, Indigenous populations, a subgroup in Australia with a disproportionate burden of TB [1], were under-represented in this study. The power and heterogeneity of the sample was such that analysis for independent predictors of default and relapse was not possible; also, any trends over the 4 years of the study in guideline adherence could not be tested due to small numbers. These questions should be addressed in further TB studies in this region.

Overall, the quality of TB management practices by comparison to published guidelines was generally good and outcomes excellent in this study. This study highlights certain quality domains, adherence to which could be emphasised in future local, state and national guidelines. While the diagnostic robustness of cases was comparable to other low-prevalence countries, the lack of laboratory evidence in a substantial proportion of EPTB cases merits further study into novel approaches to EPTB diagnosis, and calls for more standardised classification systems to improve patient care and surveillance of this increasingly common disease.

## Supporting Information

S1 Table Quality domains examined in characterisation of management practices. (DOCX)
