# Supplementary material for: Epidemiology, Diagnosis and Management of Extra-Pulmonary Tuberculosis in a Low-Prevalence Country: A Four Year Retrospective Study in an Australian Tertiary Infectious Diseases Unit
Source: PLoS One. 2016 Mar 10;11(3):e0149372. doi: 10.1371/journal.pone.0149372 (PMC4786131; doi:10.1371/journal.pone.0149372)
Supplement: S1 Table — (DOCX) [file pone.0149372.s001.docx]

| **Table S1. Quality domains examined in characterisation of management practices** |  |
| --- | --- |
| **Management practice** | **Reference Guideline** |
| Adjunctive  investigations |  |
| Perform HIV serology in all EPTB cases^a^ | [[1](#_ENREF_1)], [[2](#_ENREF_2)], [[3](#_ENREF_3)],[[4](#_ENREF_4)] |
| Screen for HBV infection in high risk patients^b^ | [[1](#_ENREF_1)], [[2](#_ENREF_2)] |
| Screen for HCV infection in high risk patients^b^ | [[1](#_ENREF_1)], [[2](#_ENREF_2)] |
| Baseline eye review (in cases on ethambutol ) | [[1](#_ENREF_1)], [[2](#_ENREF_2)],[[4](#_ENREF_4)] |
| Perform chest imaging on all EPTB cases to consider pulmonary co-infection | [[3](#_ENREF_3)] |
| Liver function indices, renal function and FBC at baseline | [[1](#_ENREF_1)],[[2](#_ENREF_2)] |
| Anti-tuberculous therapy |  |
| Standard first-line therapy whilst waiting susceptibilities | [[1](#_ENREF_1)],[[2](#_ENREF_2)],[[4](#_ENREF_4)] |
| Consider longer treatment regimens for EPTB subtypes such as CNS disease^c^ | [[1](#_ENREF_1)], [[2](#_ENREF_2)] |
| Consider referring for DOT^d^ | [[1](#_ENREF_1)], [[2](#_ENREF_2)], [[4](#_ENREF_4)] |
| Daily therapy in at least the intensive phase in HIV-TB co-infections | [[1](#_ENREF_1)],[[4](#_ENREF_4)] |
| Ensure effective ARV eventually co-administered with HIV-TB infection^e^ | [[1](#_ENREF_1)],[[4](#_ENREF_4)] |
| Consider delaying initial commencement of ARV in HIV-TB coinfection | [[1](#_ENREF_1)], [[2](#_ENREF_2)], [[4](#_ENREF_4)] |
| Caution with rifampicin in HIV-TB coinfection | [[1](#_ENREF_1)], [[2](#_ENREF_2)],[[4](#_ENREF_4)] |
| Pyridoxine supplementation (in cases receiving isoniazid) | [[1](#_ENREF_1)], [[2](#_ENREF_2)], [[4](#_ENREF_4)] |
| Steroid use in all CNS or pericardial disease | [[1](#_ENREF_1)], [[2](#_ENREF_2)], [[3](#_ENREF_3)], [[4](#_ENREF_4)] |
| Consider and manage potential drug-drug interactions | [[1](#_ENREF_1)], [[2](#_ENREF_2)], [[4](#_ENREF_4)] |
| ^a^EPTB = extrapulmonary TB |  |
| ^b^High risk patients = birth in Africa, Asia or IVDU |  |
| ^c^CNS = central nervous system, ARV = antiretroviral therapy |  |
| ^d^DOT = directly observed therapy |  |

1. Centers for Disease Control & Prevention (2003) Treatment of tuberculosis. MMWR Recomm Rep 52: 1-77.

2. Antibiotic Expert Group (2010) Therapeutic guidelines: antibiotic. Version 14. Melbourne: Therapeutic Guidelines Limited; 2010.

3. World Health Organisation (2006) Improving the diagnosis and treatment of smear-negative pulmonary and extrapulmonary tuberculosis among adults and adolescents.

4. World Health Organisation (2009) Treatment of Tuberculosis: guidelines, 4th Edition, 2009.
